# Ecological innovations in the Cambrian and the origins of the crown group phyla

**DOI:** 10.1098/rstb.2015.0287

**Published:** 2016-01-05

**Authors:** Graham E. Budd, Illiam S. C. Jackson

**Affiliations:** Department of Earth Sciences, Palaeobiology Programme, Uppsala University, Villavägen 16, Uppsala 752 36, Sweden

**Keywords:** Spiralia, Lophotrochozoa, crown group, nervous systems, gut, trace fossils

## Abstract

Simulation studies of the early origins of the modern phyla in the fossil record, and the rapid diversification that led to them, show that these are inevitable outcomes of rapid and long-lasting radiations. Recent advances in Cambrian stratigraphy have revealed a more precise picture of the early bilaterian radiation taking place during the earliest Terreneuvian Series, although several ambiguities remain. The early period is dominated by various tubes and a moderately diverse trace fossil record, with the classical ‘Tommotian’ small shelly biota beginning to appear some millions of years after the base of the Cambrian at *ca* 541 Ma. The body fossil record of the earliest period contains a few representatives of known groups, but most of the record is of uncertain affinity. Early trace fossils can be assigned to ecdysozoans, but deuterostome and even spiralian trace and body fossils are less clearly represented. One way of explaining the relative lack of clear spiralian fossils until about 536 Ma is to assign the various lowest Cambrian tubes to various stem-group lophotrochozoans, with the implication that the groundplan of the lophotrochozoans included a U-shaped gut and a sessile habit. The implication of this view would be that the vagrant lifestyle of annelids, nemerteans and molluscs would be independently derived from such a sessile ancestor, with potentially important implications for the homology of their sensory and nervous systems.

## 1. Introduction

The Cambrian explosion continues to excite interest from a wide range of biologists interested in morphological, ecological and developmental evolution. The popular view, inspired by accounts such as Gould's *Wonderful Life* [1], sees an almost instantaneous appearance of the phyla, an approach that long proved attractive to evolutionary developmental biologists (and indeed palaeontologists), while molecular clocks continue to place the origin of the animals and even the bilaterians well before the Cambrian in the Cryogenian, i.e. before 635 Ma [2,3]. In 2000, it was suggested that a more accurate view of the Cambrian explosion lay between these two extremes, with a general application of the stem- and crown group concepts to Cambrian systematics [4]. This had the effect of smearing the appearance of the extant (i.e. crown group) phyla upwards into the Cambrian and even later (e.g. the possibility that crown group annelids and echinoderms emerged in the Ordovician), without denying the likely appearance of the largest clades of animals in the (Late) Ediacaran. In the 15 years since this paper was published, it remains true—rather remarkably—that no putative crown group animal phylum member from the Ediacaran commands universal assent. Indeed, putative single occurrences of bilaterians from the Ediacaran all create considerable, but rarely acknowledged problems [5]. For there to be (for example) Ediacaran annelids, molluscs or arthropods implies that a great deal of diversification within animals had already taken place by that point, and the general lack of other suggestive fossils is thus—to say the least—somewhat peculiar. For example, if one accepts the recent assignment of the Ediacaran *Sabellidites* to the siboglinids [6] (i.e. crown group annelids [7]), then the inevitable phylogenetic conclusion is that a path from the base of the animals to a point deep within crown group Annelida has been already traced, and this implies many other important branching points have also been passed. Why, then, would only a relatively poorly preservable and highly derived member of this clade be preserved? Why would no scolecodonts [8] be recoverable from the Ediacaran (and indeed Cambrian) record, if crown group annelids had already evolved? Furthermore, why would no other indication of any other internal node in the bilaterian tree that must already have been passed be present? A variety of answers to this question have been given over the years [9–12], but they all rely on the idea that internal nodes of trees are in some way ‘different’ from external ones—and that, for example, it is permissible to claim that all features of the living phyla pertinent to large body size—coelom, blood vascular system, musculature, etc.—all happen to be independently derived within the (arbitrary) groupings that comprise the modern phyla. This is—we believe—the essential message of the several papers that have attempted to decouple phylogenetic divergence from phenotypic evolution. This view is the twin of that which claims *many* features of the extant phyla are homologous and that they have thus been lost on a sometimes grand scale in the many phyla that lack them [13]. This dismissal of the use of systematics has thus led to a great deal of unnecessary confusion in understanding the pattern of diversification in the Cambrian explosion, to which we now turn.

## 2. Stem- and crown group stability and diversification: an approach through modelling

The general pattern of the early Cambrian fossil record is becoming slowly clearer, as discussed below, with most easily fossilizable phyla appearing within the period. Despite the recognition that fossils from the Cambrian do not need to be *crown group* members of the phylum they are related to, it is nevertheless a striking feature of the Cambrian fossil record—and one that has been become clearer in the last 15 years or so—that there is indeed a fairly remarkable stability in the appearance of crown group phyla, i.e. the groupings consisting of the last common ancestor of all living members of a phylum plus its descendants [14]. Even if extraordinary innovations and perturbations of the ground-pattern of a clade occur later (for example, in pelagic holothurians [15], rhizocephalan arthropods [16] and haemoglobin-lacking fish [17]), the lesson of the Burgess Shale [18–20] and Chengjiang Biota [21] is that very important components of modern diversity were present by mid-Cambrian time (i.e. by about 515 Ma or so). This quite striking pattern raises two obvious questions. (i) Even if one accepts that many Cambrian taxa formally lie in stem and not crown groups of the phyla, why do the crown groups appear so early? Why, for example, don't the crown groups of phyla such as arthropods and annelids date back only to the Jurassic and not to the Cambrian/Ordovician? (ii) What is the biological background to this early establishment?

The first of these questions has constantly been raised over the last few decades, with the typical answers falling into two categories of restraint. The first and still popular answer is that unprecedented genetic developmental flexibility during the early stages of animal evolution gave rise to a burst of ‘experimentation’ in animal body-plan evolution, which was later at least partly closed off by increasing elaboration of genetic control systems [22]. In this view—which has evolved over several decades as a result of data from the field of ‘evo-devo’ [22–24]—the locus of evolutionary change would shift from highly conserved developmental ‘kernels' that were refractory to change, to less constrained regions of the genome [22]. This view is essentially identical to the view that Riedl had of ‘burden’ in evolution ([25,26]; see also [27] for a view of how such constraints are overcome).

The other popular answer is the so-called ‘filling of the ecological barrel’, in which constraints were imposed externally by increased niche occupancy ([28]; both hypotheses are carefully reviewed by Erwin [23]). In short, the first type of answer is that no later high-level disparity was generated; the second was that it could not survive. It is noteworthy, however, that no phylogenetic tree showing how genetic constraints evolved through time has been published [5], and this is not coincidental, for it seems impossible to square any sort of parsimonious view of animal evolution with the idea that the living phyla can coincide with such constraints—this confuses the taxonomic with the phylogenetic hierarchy, and they are not at all the same thing (phyla, classes, etc. are hierarchical with respect to the ranks above and below them, but not to each other, whereas all clades are within a hierarchical structure, irrespective of rank: a (cladistically defined) phylum can thus be sister group to a pair of other phyla).

Although some research does imply that Cambrian rates of morphological change or amounts of variation were higher than in later periods [29,30], it has however proved so far difficult to come up with absolutely decisive tests on whether this variation is genetic or ecological in nature. Several tests nevertheless have been proposed, including the challenge to the fossil record from molecular clocks, which *if true* might imply long periods of phylogenetic divergence with little detectable concurrent morphological change. A second implication might thus be that many major developmental innovations took place without major morphological change, which would seem to refute some versions of the developmental hypothesis [24]. Indeed, the discovery of the conservation of many important developmental genes across phyla has led to a shift away from considering the establishment of major developmental structures as causal in the Cambrian explosion [22].

Similarly, the study of biotas either side of mass extinctions such as the end Permian example have been used to examine the filled-ecological theory [23] although even such profound extinction events as this may not fully reflect the unique situation as animals evolved for the first time. Nevertheless, the basic data under examination here—i.e. the very meaning of morphological variation in the fossil record—given that there can be a strong control by the environment on the phenotypes the genotype generates, requires more subtle investigation than has been hitherto accorded to it. Finally, a very popular current view of the Cambrian explosion, although not one that has been fully fleshed-out with regards to mechanism, is that the unique physical conditions of the Earth during this interval created a unique interval for ‘body-plan innovation’ [31]. Of these, perhaps the most interesting are those relating to ‘niche construction’, whereby organisms themselves modify the environment in a way that generates more diverse ecological opportunities for their successors [32–34].

**Figure 1.**
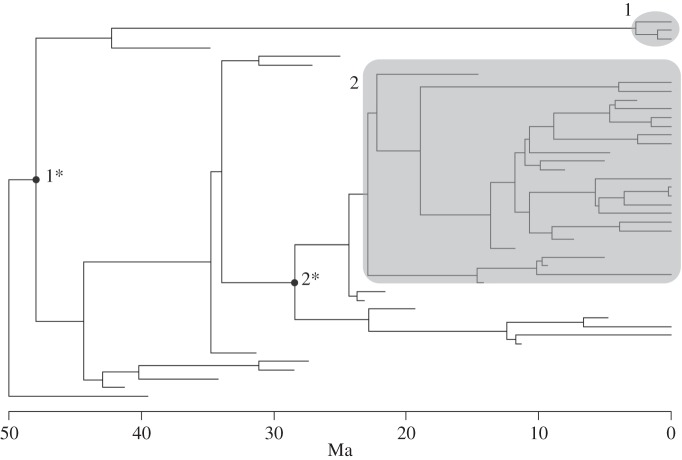
A typical simulation of a tree with a birth–death model (see text for details). Typical crown groups (numbered) are marked in grey; the divergence time of the total groups that generated them is marked with the respective asterisked number. Note that the time scale is arbitrary and that patterns of diversification over long periods of time may not be accurately reflected in simulations [35], although this will not affect the results herein.

The timing of the emergence of crown groups relative to the total groups that encompass them is necessarily a hierarchical process, like all other phylogenetic ones. What governs this process is, however, unclear. For example, it may be that absolute rates of speciation or extinction, or the rates of diversification, control when crown groups emerge. In order to investigate the mechanics of crown group emergence, we created a simple model of birth–death processes in time series (figure 1) to examine what controlled the relative timing of crown group emergence (i.e. the time when the last common ancestor of all *living* forms emerged within a particular total group). A birth–death model begins with a lineage splitting in two and from then on allows that speciation occurs at a certain rate and extinction at another. So, in each time step of a simulation, there is a certain likelihood of a lineage speciating and a certain likelihood of it going extinct. Changing these rates alters the structure of the generated trees in different ways. For example, high speciation rates obviously generate denser trees. Most studies of this sort of tree dynamics focus on diversification rates, which is the extinction rate subtracted from the speciation rate (e.g. [36,37]). Ricklefs [37] also identifies biases of which one should be aware when estimating diversification rates based on phylogenetic reconstructions, the common method of estimating historic diversification rates employed today (cf. [38]).

For all simulations, the R package *TreeSim* [39] was used with the R software, v. 2.13.2. (see [40] for algorithms used in this package). All graphs were generated in Microsoft Excel, v. 14.0.4734.1000. To investigate the scenarios of equal rates of speciation and extinction, 50 trees of 114 time steps were generated with equal extinction (*E*) and speciation (*S*) rates 0.1, 0.3, 0.5, 0.7 and 0.9. Sets of 100 trees were generated with each set of parameters. Trees that went extinct before the present were discarded. The crown group emergence times of the first 50 trees surviving to the present were saved.

Owing to computational limitations, trees generated where *S* exceeded *E* could not be longer than 15 time steps. The diversification rate (*D*) is *S* − *E*. Consequently, to investigate the other scenarios, *E* was set to 0.1, 0.3, 0.5, 0.7 and 0.9. For each *E*, 50 trees each were simulated for *D* values of 0, 0.2, 0.4 and 0.6. When *D* = 0.8, i.e. with *S* = 0.9 and *E* = 0.1, the simulations could not be run owing to computational difficulties. In those instances where *S* > 0.9, trees were not generated. Sets of 100 trees were generated with each set of parameters. Again, the trees that did not survive to the present were discarded and the crown group emergence time of the first 50 surviving trees was saved.

### (a) Results

**Figure 2.**
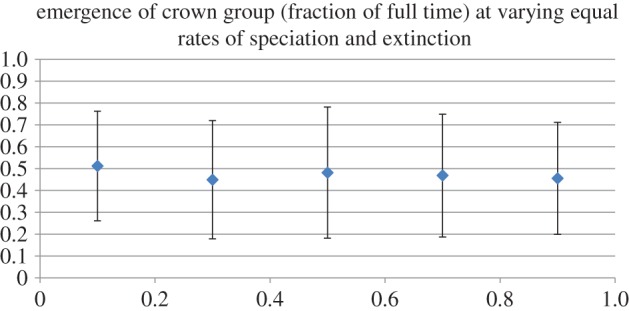
Results of simulations of varying speciation (*S*) and extinction (*E*) rates where *S* = *E* (i.e. where diversification rate (*D*) = 0): error bars = ±2 s.d. *y*-axis represents fraction of total time at which crown group arises; *x*-axis represents *S*/*E*.

**Figure 3.**
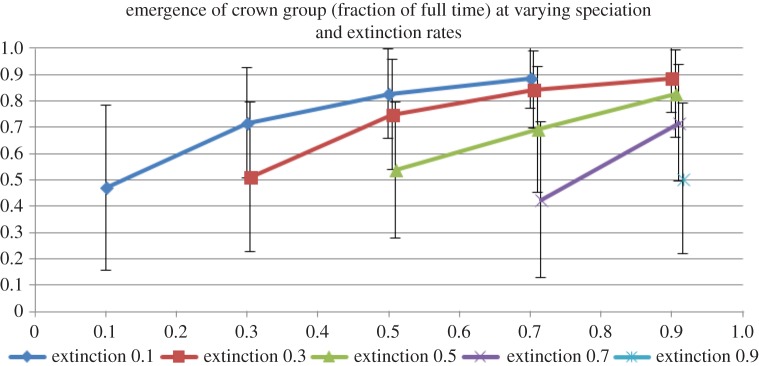
The effect of increasing speciation relative to extinction rates on the time of emergence of crown groups relative to their total groups (*y*-axis). Each line represents an extinction rate with the *x*-axis providing the corresponding speciation rate. Note that some values are slightly offset from their *x*-value to avoid blurring of error bars; the only real positions on the *x*-axis are 0.1, 0.3, 0.5, 0.7 and 0.9.

**Figure 4.**
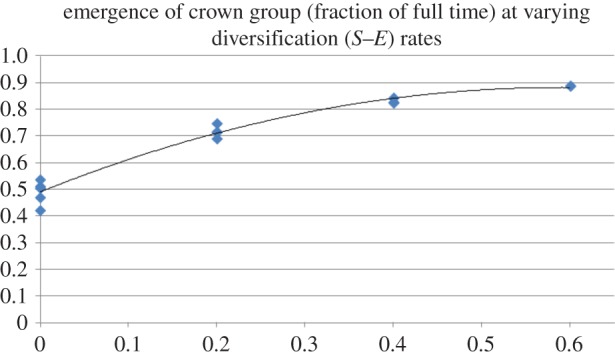
The effect of increasing diversification rates (*x*-axis) on the emergence time of crown groups relative to the total group (*y*-axis). The data points used here are the values from figure 3.

When extinction and speciation rates were set to be equal, the crown group tended to emerge at 50% of the time of the age of its total group, no matter what the absolute values were of the rates (figure 2). Setting extinction rates to be higher than speciation rates did not, as can be expected, tend to lead to trees robustly surviving for the duration that the simulation ran. However, when speciation rates were set at rates successively higher than extinction rates (i.e. in scenarios with various positive diversification rates), the crown groups emerged closer and closer to the origin of the respective total groups as diversification rate increased (figures 3 and 4).

### (b) Discussion

Irrespective of what happens in subsequent crown group diversification, the crown group itself emerges within the total group at (the latest) 50% of the time of diversification of the total group, when rates are kept constant throughout the history of the clade. As diversification rates increase, the origin of the crown group closely approaches that of the total group. Although we have not as yet modelled crown group dynamics when *D* varies throughout its history, these results suggest that the early emergence of crown groups is simply an inevitable consequence of high diversification rates, at least early in a clade's history. This is of particular relevance to the Cambrian explosion, because recent studies indicate that Cambrian rates of evolution in both morphology and molecules may have been considerably elevated relative to later periods of time [29,41].

An interesting corollary of this result is that, with uniform rates of diversification, it is very difficult to develop temporally long stem-groups, for this would imply that there would have to have been extended periods of negative diversification (which would in turn make survival of the clade to the present to generate an extant crown group extremely unlikely). Without the effects of fluctuations in diversification rate, then, it is very unlikely that the total groups of today's phyla emerged enormously before the crown groups did—another reason to think that they also in fact emerged around the beginning of the Cambrian. Many different models exist for diversification patterns and their effect on phylogeny (reviewed in e.g. [38]), and a popular view of major diversification would be a ‘waxing–waning’ model, where diversification rates are initially high and then decline through time (fig. 1 of [38]; but see also [42] for documentation of the reverse pattern in post-Palaeozoic echinoids). What effect this would have on crown (relative to total) group appearance is rather unclear, although it would obviously tend to make crown groups on average appear later than with uniform (and high) diversification rates (cf. fig. 1(3) of [38]). Strathmann & Slatkin [43] investigated some effects of varying rates on clade persistence and concluded that an initial burst of diversification in the Cambrian would confer a considerable protective effect on clades from later extinction, especially given realistic levels of diversity. With uniform diversification rates, the total/crown group divergence ratio should be scale-invariant, which avoids the problem of singling out ‘major’ groups (i.e. phyla) for investigation. With declining diversification rates through time, one should expect that total groups that arise early would have shorter stem-groups than ones that arise later, and perhaps this might be reflected in the taxonomic hierarchy to some extent (i.e. one would expect phyla to have shorter stem-groups than, say, families).

As far as patterns of stability and appearance are concerned, these results strongly suggest that crown groups in general appear soon after their total groups, with the consequence that all features of modern groups appear early. Rather than being a consequence of early developmental flexibility [23] that later closed off, crown groups simply must appear early if they are to be crown groups (i.e. have a chance of surviving to this day). Morphological features associated with the crown group as a whole (including, naturally, all aspects of the nervous system) must thus also have appeared by this time. This pattern (cf. [44], a strangely neglected paper that comes to substantially the same conclusions) suggests that the interesting aspect to the crown group phyla is not their relatively early appearance, but whether or not they remain static later in the Phanerozoic. Raup [44] ponders the striking rapidity of morphological divergence that leads to such groups and suggests that this is a biological rather than non-biological phenomenon. However, we suggest that this rapidity too is a (rather unexpected) artefact, or rather a selection effect: it is precisely those groups that survived to the present that must also have had a rapid rate of morphological divergence. This could in principle be tested by examining groups that did not survive to form a modern crown group—the striking prediction here would be that these groups would have slower rates of initial morphological diversification, and thus had less chance of avoiding extinction before the present.

This thus raises a subtle and important question: did the extant phyla all diversify rapidly at around the same time because they are all total-group animals, and given their survival must have appeared rapidly after their total group did, or does this apparently correlated rapid diversification imply a true ecologically mediated event? The issue is not that there is an incompatibility between, for example, adaptive radiations and the pattern presented here, but rather that there is a selective effect biased towards the survival of rapid radiators. Within an event such as the Cambrian explosion, where it seems likely that rapid evolution was ongoing, some taxa even so radiated slowly; but the most rapid radiators would be most likely to survive: the establishment of a standing diversity is essential for clade longevity, and once generated is likely to be robust [43] to at least stochastic variation. This will mean that extant taxa are a biased sample of evolutionary rates within radiations, without denying the fact that radiations do in fact exist. A comparative approach between radiations of different ages (e.g. those of mammals or angiosperms) might also be of interest to investigate if age of surviving (i.e. crown group) origins varies through time: one prediction might be that younger radiations have more variable crown group dates of origin (and speeds of diversification).

Finally, the question of known crown group phyla that appear to have long stems and short crowns should briefly be addressed. The most obvious example of this is the Ctenophora, total group members of which date apparently back to the Ediacaran [32,45], although crown members apparently diverged much more recently [46]. These exceptions to the rule emphasize that a statistical approach needs to be taken and that even old and well-established groups have a chance of going extinct (or nearly so), with the trilobites and Palaeozoic echinoids being respective examples [47]. Both the extremely rapid radiation and final extinction of a group such as the trilobites incidentally strongly suggest that the fates of groups are not purely stochastic ([35]; cf. ch. 7 of [48]).

We now turn to the Cambrian fossil record itself to interrogate its utility in establishing the order and ecological significance of these early clades.

## 3. Cambrian worlds: the sequence of faunal change in the Cambrian

**Figure 5.**
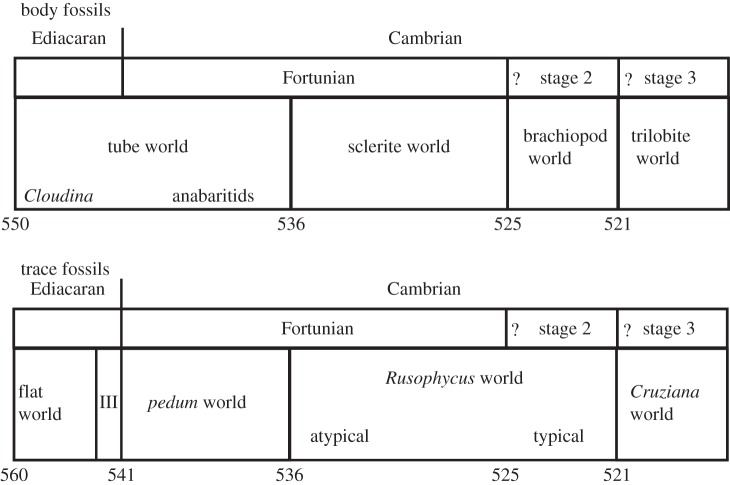
Representative intervals of Cambrian time for trace and body fossils (see text for details). The first two trace fossil intervals represent ‘Proterozoic II’ and ‘Proterozoic III’ of Jensen [51], the last the Lower Cambrian zones of Macnaughton & Narbonne [52].

**Figure 6.**
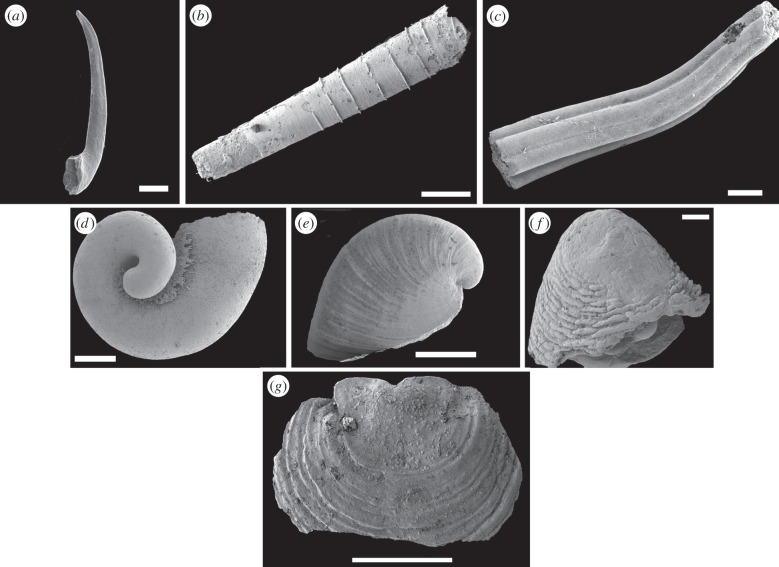
Representative early Cambrian (spiralian?) fossils, with the oldest at the top. (*a*–*c*) ‘Tube world’ fossils appearing before about 536 Ma. (*a*) The protoconodont (total-group chaetognath) *Prothertzina unguliformis*, Kuanchuanpu Formation, China. (*b*) *Anabarites tristichus*, Siberia. (*c*) *Anabarites hexasulcatus*, Siberia. (*d*–*f*) ‘Sclerite world’ fossil lophotrochozoans (presumably total group molluscs) from about 536 Ma onwards. (*d*) *Barskovia hemisimmetrica*, Tajmyr, Siberia. (*e*) *Anabarella plana*, Bol'shaya Kuonamka, Siberia. (*f*) *Purella antiqua*, Bol'shaya Kuonamka, Siberia. (*g*) A ‘brachiopod world’ representative, *Tumuldaria incomperta*, a very early paterinid brachiopod from the Pestrotsvet Formation of Siberia [61]. Scale bars, (*a*,*d*) 200 µm, (*b*) 500 µm, (*c*) 300 µm, (*e*) 2 mm, (*f*) 100 µm and (*g*) 1 mm. Photocredits, (*a*) Jean Vannier, (*b*–*f*) Artem Kouchinsky and (*g*) Christian Skovsted.

Although correlation of the lower part of the Cambrian remains controversial [49,50], the advent of relatively high-precision chemostratigraphy and improved biostratigraphic information has yielded a somewhat clearer picture of the diversification that took place in basal Cambrian times (figure 5) [53]. Rather surprisingly to the non-specialist, the basal parts of the Cambrian show rather sparse diversity, dominated by an assemblage of tubes (of uncertain affinities), notably *Anabarites*, and a moderately diverse trace fossil assemblage. Other very basal fossils include the protoconodont *Protohertzina*, sponge spicules and rather modern-looking ctenophores [54]. From very close to the Ediacaran–Cambrian boundary comes a recently described sclerite [55] that shows distinct similarities to those associated with ecdysozoans such as priapulids (a basal Cambrian scalidophoran body fossil, *Eopriapulites*, has also been reported from the Kuanchuanpu Formation of South China [56]). This ecological assemblage, which might be labelled ‘tube world’ (figure 6*a–c*), shows some similarities to that of the terminal Ediacaran, which is also characterized by a series of tubes, most famous of which is *Cloudina*, although a variety of other forms, some undescribed, are also known.

Depending on dating and correlation of sections such as in Mongolia, where there are hints that some cryptic discontinuities exist (e.g. [46,57]), the next clear event in the fossil record is perhaps at about 536 Ma, when more diverse small skeletal fossil assemblages start to appear [53,58]. These comprise various cap-shaped fossils including the ‘scaly’ shells *Purella* and *Maikhanella* [59,60], halkieriids and many other taxa traditionally associated with the Tommotian in Siberia. This ‘sclerite world’ (figure 6*d–f*) gives rise, at around the base of the traditional Tommotian, approximately 525 Ma, to assemblages more dominated by (for the first time) brachiopods (figure 6*g*) and hyolithids, with archaeocyathids and associated biota also entering around this time [62] in carbonate environments. Finally, by about 521 Ma or so, trilobites appear in the record, and undergo a rapid diversification, so that by end-Lower Cambrian time at around 510 Ma, some 590 genera are recorded (numbers extracted from [63] plus survey of later literature), a diversification paralleled by other groups of total-group arthropods [64]. We know also that by this time, many of the macroscopic phyla, at least as total groups, are also present [65].

A similar pattern of increasing diversification is also seen in the trace fossil record [51,66]. Three Late Ediacaran assemblages, from approximately 560 Ma onwards, show a sequence from first simple two-dimensional traces, through to the first hints of three-dimensionality and then more clear, *Treptichnus*-like examples from just before the Ediacaran–Cambrian boundary. Within the Cambrian, the moderately diverse assemblage of broadly treptichnid taxa such as *Treptichnus* itself and *Didymaulichnus* [67] are joined by *Rusophycus* (the earliest ones rather atypical) followed by *Cruziana* at around the same time as the trilobites.

## 4. Ecological diversification in the Cambrian

The Cambrian fossil record before the oldest major exceptionally preserved biotas (at perhaps around 515 Ma) is frustratingly fragmentary, because it is an interval of time that despite its sparseness must represent a major animal radiation [68]. Nevertheless, despite its obvious incompleteness, the subtle clues it provides give some shape to the inferred animal radiation that lies behind it. Perhaps the most significant taxon is *Protohertzina* and allied taxa, which are recovered from extremely early Cambrian beds around the world, and which on a variety of grounds (including soft-part preservation from the Chengjiang biota) can be very reasonably considered to be total-group chaetognaths [69]. The precise phylogenetic position of the chaetognaths is uncertain, but their most likely position is as a sister group to all other spiralians or perhaps even all other protostomes [70], which corresponds well to such an early appearance, which may even predate the first sclerites [58]. Although the interpretation of the ecology of these early chaetognaths must remain somewhat uncertain, their close morphological similarity to modern examples strongly suggests that they were pelagic and predatory, with a pelago-benthic ecology perhaps being most likely. Although modern chaetognaths are normally considered to be (voracious) predators, the provocative claim has also been made that they largely live off dissolved organic matter [71], at least in the wild; even if this is untrue, many aspects of chaetognath feeding remain unclear. One possibility, despite their appearance, is thus that early chaetognaths mostly fed off the higher concentrations of dissolved organic matter and bacteria near the sea floor [72], and perhaps opportunistically preyed on the benthos to supplement this.

This early hint of invasion of the planktonic realm is followed, somewhat later, by more widespread remains of microscopic arthropods [73], suggesting that their modern dominance here started early. However, even though body fossils of arthropods are not known until the first trilobites, early trace fossils (including at the very least *Rusophycus*, and perhaps also *Monomorphicnus*) strongly suggest that limb bearing total-group arthropods had emerged only a few million years after the beginning of the Cambrian. Similarly, both the trace fossil and (increasingly) the small carbonaceous fossil record [73] suggest that scalidophoran ecdysozoans had emerged by the opening of the Cambrian [55]. The relationship of such taxa to the arthropods is, however, somewhat unclear, given that cycloneuralians may be paraphyletic relative to the arthropods [74].

The spiralians and the lophotrochozoans (here considered to be a subgroup of the spiralians minimally containing brachiopods, annelids, molluscs and phoronids, possibly with the addition of nemerteans and the so-called ‘Polyzoa’) within them seem to be well represented by the sclerites known from about 536 Ma [58]. Even though many of these early sclerites are problematic and cannot be assigned with confidence to particular lophotrochozoan crown groups, they clearly fall within this clade. Their absence from before this is somewhat noteworthy (see below). Conversely, although some early Cambrian trace fossils were potentially made by enteropneust-like deuterostomes, deuterostomes do not emerge in the body fossil record until the first echinoderms from around 520 Ma [70]. Putting these first occurrences together allows a reasonable picture to be built up of the timing of early Cambrian bilaterian radiations (see fig. 1 of [5]; fig. 3 of [70]), even accepting the very significant preservational shortcomings of this interval. In particular, the trace fossil record, which is considerably less likely to be subject to the sorts of negative bias that affect soft-bodied fossils, suggests that (stem-group) bilaterians started to appear at around 560 Ma and that there is an interval of perhaps 15–20 Ma before the first suggestions of crown group bilaterians in the guise of more complex traces and the first likely bilaterian body fossils.

Several features stand out in this reconstruction, of which the most interesting is perhaps the lack of clear spiralian taxa from before *ca* 536 Ma, a lack that may also partly extend to the trace fossil (in other words, in addition to the lack of obvious lophotrochozoan body fossils in the very basal Cambrian, most earliest Cambrian trace fossils seem closest to ecdysozoan ones, although some might also be deuterostome). What, then, was the ecology of the earliest spiralians?

## 5. A ‘U-tube’ theory for early lophotrochozoan ecology

**Figure 7.**
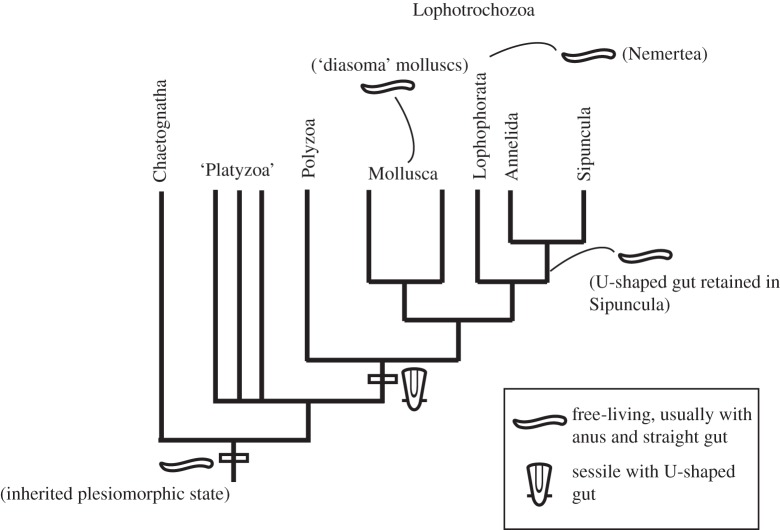
The phylogenetic scenario for spiralian evolution envisaged by the ‘U-tube’ theory in an illustrative spiralian phylogeny. Some taxa are grouped, so that ‘Lophophorata’ = Phoronida + Brachiopoda; ‘Polyzoa’ = Cycliophora, Ectoprocta and Entoprocta; ‘Platyzoa’ = the various smaller taxa (including e.g. Gnathostomulida and Syndermata) treated by Laumer *et al*. [78]. Some of these groups, especially the Polyzoa, are controversial, but their absence may not materially affect the argument herein. The position of Nemertea is unclear, but they may be the sister group to Lophophorata [78].

It is usually considered likely that a straight through-gut, mouth and anus are homologous throughout the bilaterians, even if the evidence for this is not as strong as might be thought, with the anus especially having a very chequered history [75]. These features are mostly present in the deuterostomes, ecdyosoans and basal spiralians such as chaetognaths. However, one striking feature about the spiralians, and especially the lophotrochozoans, is the widespread occurrence of a sessile ecology and (often associated) U-shaped gut (table 1, see also fig. 1 of [75]). While not too much attention has been paid to this phenomenon, with the implied supposition that these U-shaped guts are all convergences, we believe, on phylogenetic grounds, that serious consideration should be given to the opposite view, which is that especially lophotrochozoan U-shaped guts are potentially homologous (*qua* U-shaped guts). Like all theories of homology, this one too should be ultimately grounded in phylogeny (figure 7). However, the spiralians are notorious for having an only poorly resolved phylogeny (e.g. [70,76–79]), so there is no stable phylogeny to test this idea against at present. Nevertheless, most analyses imply that there is a monophyletic Lophotrochozoa, which may include, as well as the taxa named above, the entoprocts, ectoprocts and cycliophorans (the ‘Polyzoa’) and the nemerteans as particularly surprising members; and, against this, a rather heterogeneous assemblage of smaller spiralians including taxa such as the syndermatids (rotifers plus acanthocephalans), platyhelminthes (flatworms) and gnathostomulids. This latter assemblage has recently been argued to be paraphyletic (as opposed to forming a monophylum, the Platyzoa) and it is thus possible that the lophotrochozoans arose from within such an assemblage (figure 7; [78]).

**Table 1.** Gut shape and whether sessile or not for most of the spiralian phyla. In groups where mixed states are known, the most likely ground plan is given (e.g. annelids). See also [75] and text for details.

taxon	sessile?	U-shaped gut?
Mollusca	no	yes?
Annelida	no	no
Sipuncula	no	yes
Phoronida	yes	yes
Brachiopoda	yes	yes
Nemertea	no	no
Entoprocta	yes	yes
Ectoprocta	yes	yes
Cycliophora	yes	yes
Syndermata	no	no
Micrognathozoa	no	no
Platyhelminthes	no	no
Gastrotricha	no	no
Chaetognatha	no	no

Assessing the nature of the gut and sessile versus vagrant ecology in the spiralians is not totally straightforward, partly because of uncertainties in the phylogenies of key taxa (in particular the Mollusca [79]). The old concept of Diasoma (through-body) versus Cyrtosoma (humped-body) is nevertheless perhaps still of relevance here [80]. Gastropods and cephalopods (and essentially scaphopods (see, for example, comments in [81]) all have U-shaped guts, and given the difficulties in recovering molluscan phylogeny, it is possible that this state characterizes the ground plan of the crown group, as would be implied especially by analyses that place the polyplacophorans within conchiferans [82]. If the Cambrian helcionelloids are, as argued cogently by Peel [83], not closely related to the living monoplacophorans, then the state of their gut must be currently unclear and might (given their generally high-conical morphology [60]) also be U-shaped. The gut of polyplachophorans is straight, but their relationships with the conchiferan taxa remains unclear and they may be an ingroup as sister group to the monoplacophorans [82].

The presence of sessile/tube-dwelling lophotrochozoans with U-shaped guts is considerably enhanced by the Cambrian fossil record, which as well as yielding well-known taxa with U-shaped guts such as the hyolithids and orthothecids [84,85] has also generated a number of very intriguing taxa that appear to be situated in or close to the stem-group of the brachiopods. These include the stem-rhynchonelliform *Longtancunella* [86,87] that seems to show that the U-shaped gut is plesiomorphic within the brachiopods, and *Yuganotheca*, an even more remarkable form suggesting that stem-group brachiopods possessed an (in this case) agglutinated tube [77,88]. In addition to these taxa, the tommotiids *Eccentrotheca* and *Micrina* have been shown or suggested to be effectively tube-dwelling [89,90]. Other pertinent taxa may include *Cotyledion*, which was described as a (very large) stem-group entoproct, but could lie even deeper in the stem-group of the Polyzoa (the putative clade consisting of entoprocts, ectoprocts and cycliophorans) and which possesses sclerites [91].

While the evidence is thus still inchoate, one suggestion must be that the lophotrochozoans (including here the Polyzoa) all arose from sessile and tube-dwelling taxa that possessed sclerites or even frankly mineralized tubes. This is in contrast to the usual (but perhaps phylogenetically poorly supported) assumption that tube-dwelling has always been convergently acquired [92]. Although the paraphyletic assemblage of spiralians (including the chaetognaths) that they arose from all had straight guts including sessile ones (and several, it seems, had jaws), and some were mobile, it is notable that most living examples are tiny (one notable exception being the various large flatworms). It is possible that they invaded the meiofauna (or adapted other lifestyles that necessitated tiny body size) early on in the Cambrian. Whether or not the meiofauna was populated early in the Cambrian is thus a question of considerable importance in understanding early animal evolution and ecology, although as yet little direct evidence exists one way or another (for the first report of Cambrian microscopic loriciferans, see [93]; for a Cambrian tardigrade, see [94]). The suggestion is thus that the very varied assemblage of tubes (possibly even including latest Ediacaran examples such as *Cloudina* as well as anabaritids [95,96], *Ladatheca* [97], *Hyolithellus* [98], hyolithids [99], orthothecids [84] and several other forms (see partial review in [98]) represent stem-group lophotrochozoans in various positions.

Such a view of lophotrochozoan evolution is, of course, not without its difficulties. First, although these taxa certainly require much more (comparative) study, they clearly differ markedly from each other. For example, the tube structure of *Anabarites* and *Cloudina* are not at all similar [95,96,100], although this might not necessarily be expected from such a paraphyletic assemblage. Secondly, given the diversity of tube-forming taxa today, there is no reason to think that all tubes found around the Ediacaran–Cambrian transition are homologous (for example, some are likely to be forams [101]). Thirdly, not all tube-dwellers need to possess a U-shaped gut. For example, *Hylolithellus* has been suggested to possess a straight gut like the chaeopterid polychaetes [86,98]. Finally, some key taxa have been described that are not tube-dwelling but yet seem to have lophotrochozoan features. These include in particular the halwaxiids [90,102] (and, more generally, the coeloscleritophorans) which appear in the early wave of small skeletal fossil appearances, which are known (in at least some cases) to be elongate and mobile slug-like organisms with (presumably) a straight gut. The halwaxiids have continued to be problematic despite various attempts at placing them in one or other group [103–106], although their overall morphology is clearly suggestive of the lophotrochozoans. Other taxa that may be of relevance here include the enigmatic bivalved stenothecoids such as *Stenothecoides* and *Bagenovia* [107,108] and the more well-known rostroconchs such as *Watsonella* [109]. Kouchinsky [107], for example, suggested that the stenothecoids were the end-result of a loss of the trunk sclerites in a *Halkieria*-like animal, although the process could also have taken place in the opposite direction.

Other taxa that should (in this hypothesis) be tubular but have been suggested to be slug-like are *Camenella* and the probably related tommotiids such as *Lapworthella* and *Dailyatia* [110,111], and Skovsted *et al.* [111] consider this state to be characteristic of ‘most stem lophotrochozoans', with reference to the halwaxiids. However, in the absence of any definitive evidence of the scleritome of these (basal?) tommotiids, this reconstruction must be considered to be at present unproven.

The most problematic taxa in this theory are thus the annelids, which appear in the Lower Cambrian Sirius Passet fauna resembling vagrant polychaetes [112,113], although all the Cambrian ones are demonstrably stem-group forms [4,114]. The straight gut of annelids would in this view be a reversion to the more basal character state. Of particular interest here, though, are the sipunculans. Although they have traditionally been considered to be allied to the molluscs, molecular data have rather decisively associated them with the annelids: indeed, some studies have suggested they are in-group annelids [115–118]. If so, the Cambrian fossil record of annelids is problematic as the Chengjiang biota yields a sipunculan, whereas no crown group Cambrian anneldis are known. If sipunculans are sister group to the annelids [117], then their U-shaped gut but free-living habit may conceivably reflect an intermediate state in the loss of the sessile habit in the lineage leading to annelids *s.s.*+sipunculans. Finally, a taxon that has consistently fallen within the Lophotrochozoa is the Nemertea, which also possess a straight through-gut [119]. Their position as sister group to the brachiopods (even to the exclusion of the phoronids) is, from a morphological perspective, problematic. However, if this is their true position (which more recent publications cast doubt on [78]), then their straight gut must also be derived, irrespective of the broader conclusions of this paper.

## 6. Discussion: early bilaterian evolution and its implications for the nervous system

As discussed elsewhere [13], any attempt to reconstruct the morphology of nervous systems in the absence of direct preservation (which is extremely rare [120]) is bound to be fraught with difficulties, and must rely largely on phylogenetic considerations. The presence of ecdysozoans by very early in the Cambrian at the latest, and the appearance of various lophotrochozoan sclerites by about 536 Ma both suggest a rapid bilaterian radiation at the end of the Ediacaran, a rapidity that was, in retrospect, inevitable given bilaterian survival to the present day (see above). Although a variety of trace fossils are known from the very early period of the Cambrian, many or most can be assigned to the ecdysozoans or perhaps deuterostomes, and the relative lack of possible lophotochozoan traces is striking, given that at least stem-group lophotrochozoans are very likely to have been present at this time. One possible solution to this pattern is that after an initial, very early spiralian radiation (represented by protoconodonts in the fossil record and by the various micro-fauna spiralians of today), the stem-group lophotrochozoans adopted a sessile habit with a U-shaped gut. Both the conventional and exceptional body fossil record in the Cambrian yields a variety of tubular taxa or taxa with a U-shaped gut, most of which do not fit into crown group phyla. A detailed comparative examination of the many Cambrian tubular fossils that are known may thus yield valuable phylogenetic information that has hitherto been overlooked. Despite their varied mineralogy and structure, the great diversity known in extant taxa in form and mineralogy (e.g. [121,122]) must give impetus to the search for broader synapomorphies among the relevant clades [123] than typological views of the phyla would allow (for a recent phylogenomic perspective on mollusc–brachiopod links in this regard, see [124]).

Nervous system structural homologies are likely to have been affected by such ecological diversification early on in bilaterian evolution, in particular transitions from sessile (and filter feeding?) ecologies [92] to more active and often predatory lifestyles such as those of errant polychaetes. This may have important implications for especially aspects of brain evolution (such as the origin of the mushroom bodies and their potential homology across different clades [125,126]), because it is likely that such brain architecture, and its retention, is strongly influenced by ecology. This view is in line with that expressed elsewhere [13], that homology statements must rely on phylogenetic support as complemented by an as detailed as possible ecological and evolutionary history of the clades in question.
